# Prophylactic Extended-Field Irradiation for Patients With Cervical Cancer Treated With Concurrent Chemoradiotherapy: A Propensity-Score Matching Analysis

**DOI:** 10.1097/IGC.0000000000001344

**Published:** 2018-08-29

**Authors:** Weiping Wang, Xiaoliang Liu, Qingyu Meng, Fuquan Zhang, Ke Hu

**Affiliations:** Department of Radiation Oncology, Peking Union Medical College Hospital, Chinese Academy of Medical Sciences & Peking Union Medical College, Beijing, China.

**Keywords:** cervical cancer, extended field radiation therapy, para-aortic lymph nodes, survival, toxicity

## Abstract

**Objective:**

The aim of the study was to evaluate the efficacy and toxicity of prophylactic extended-field radiation therapy (RT) for cervical cancer patients treated with concurrent chemoradiotherapy (CCRT).

**Methods:**

Records of patients with cervical cancer without para-aortic metastatic lymph nodes who were treated with definitive RT or CCRT between January 2011 and December 2014 were reviewed. Patients were classified into the pelvic RT and extended-field RT groups. An additional dose of 50.4 Gy in 28 fractions was delivered to para-aortic lymph node regions for patients in the extended-field RT group. Cox regression and propensity-score matching (1:1) were used to compare the overall survival (OS), disease-free survival (DFS), distant failure, and para-aortic lymph node failure (PALNF) between the pelvic RT and extended-field RT groups.

**Results:**

A total of 778 patients were analyzed. Of them, 624 patients were treated with pelvic RT and 154 patients received extended-field RT. The median follow-up period was 37.5 months. In multivariate analysis, extended-field RT was an independent prognostic factor of distant failure (hazard ratio [HR] = 0.49, 95% confidence interval [CI] = 0.26–0.90, *P* = 0.023) and PALNF (HR = 0.012, 95% CI = 0.00–0.49, *P* = 0.019). However, it was not significant in predicting OS (*P* = 0.546) and DFS (*P* = 0.187). With propensity-score matching, 108 pairs of patients were selected. The 3-year OS, DFS, local control, distant failure, and PALNF rates in the pelvic RT and extended-field RT groups were 87.1% and 85.7% (*P* = 0.681), 71.0% and 80.6% (*P* = 0.199), 86.6% and 85.0% (*P* = 0.695), 21.7% and 7.0% (*P* = 0.016), and 6.6% and 0% (*P* = 0.014), respectively. The incidences of grade 3 or greater chronic toxicities were 3.5% and 6.5% in the pelvic RT and extended-field RT groups, respectively (*P* = 0.097).

**Conclusions:**

Prophylactic extended-field RT was associated with decreased distant failure and PALNF and showed a trend in improving DFS in patients with cervical cancer treated with CCRT.

## Abstract 2

Supplemental digital content is available in the text.

Lymph node metastases are the most important transport pathway of cervical cancer. The lymphatic spread in cervical cancer follows a predictable and orderly pattern from the lower to the upper regions of the pelvis, and skip metastases are rare.^1^ Para-aortic lymph nodes play an important role in the metastases of cervical cancer. It was reported that approximately 15% of patients with cervical cancer had para-aortic metastatic lymph nodes (MLNs).^2^–^4^

For patients with locally advanced cervical cancer treated with definitive radiation therapy (RT), pretreatment lymphadenectomy is not widely used. The para-aortic lymph nodes are assessed with imaging modalities in most patients. However, the sensitivity of imaging modalities is comparatively low. In the detection of para-aortic MLNs, the sensitivities of computed tomography (CT), magnetic resonance imaging (MRI), and positron emission tomography/CT (PET/CT) were just 68%, 54%, and 73% to 81%,^5^,^6^ respectively. With these imaging modalities, a considerable proportion of occult para-aortic MLNs could be missed. Prophylactic extended-field RT has therefore been used for the treatment of occult para-aortic MLNs in patients with locally advanced cervical cancer. After concurrent chemoradiotherapy (CCRT), distant failure is comparable with local recurrence and becomes the main failure pattern.^7^,^8^ Prophylactic extended-field RT may reduce the distant failure rate.

In the era of single RT, several large randomized control trials compared pelvic RT and extended-field RT for patients with cervical cancer without para-aortic MLNs. Radiation therapy oncology group 79-20 reported that extended-field RT was associated with improved overall survival (OS) and distant failure.^9^ In a controlled clinical trial of the European Organization for Research on Treatment of Cancer, prophylactic extended-field RT reduced the risk of distant failure and para-aortic lymph node metastases.^10^ Currently, the standard treatment for patients with locally advanced cervical cancer is CCRT. In the era of CCRT, studies to compare pelvic RT and prophylactic extended-field RT are limited,^11^–^15^ and the role of prophylactic extended-field RT has not been clearly established at present. In this study, we compared the efficacy and toxicity of prophylactic extended-field RT and pelvic RT for patients with cervical cancer treated with CCRT.

## MATERIALS AND METHODS

### Patients

After obtaining institutional review board approval from Peking Union Medical College Hospital, records of patients with cervical cancer treated with definitive RT or CCRT in our hospital between January 2011 and December 2014 were retrospectively reviewed. The inclusion criteria were as follows: histologically proven cervical cancer, International Federation of Gynecology and Obstetrics (FIGO) stage IB–IVA, and no evidence of para-aortic MLNs. Patients with previous surgery or RT were excluded from this study.

Pretreatment examination included gynecological pelvic examination, tumor biopsy, squamous cell carcinoma (SCC) antigen measurement, chest and abdomen CT, pelvic MRI, and CT. Some patients received PET/CT.

### Treatment

As described previously,^7^,^8^ all patients were scheduled to receive external beam radiation therapy and intracavity brachytherapy. The external beam radiation therapy was delivered with volumetric modulated arc therapy or helical tomotherapy. Gross tumor volume (GTVnd) and clinical target volume (CTV) were delineated on CT simulation images. The GTVnd was defined as pelvic MLNs. For patients treated with pelvic RT, CTV included the gross tumor, GTVnd, cervix, uterus, upper part of the vagina, parametrium, and pelvic lymph node regions (including the common iliac, external iliac, obturator, internal iliac, and presacral lymph node regions), with a superior border of the aortic bifurcation. In our institute, prophylactic extended-field RT was recommended for patients with common iliac MLNs, bilateral pelvic MLNs, and stage IIIB disease. For patients treated with extended-field RT, the CTV covered the para-aortic lymph node regions and the CTV of pelvic RT. Para-aortic regions encompassed the area adjacent to the aorta and inferior vena cava, with a lower border of the aortic bifurcation. The upper border of the extended field was usually at T12 or the renal vessel. Planning GTVnd (PGTVnd) was defined as the GTVnd plus a margin of 5 mm. Margins of 8 to 10 mm for volumetric modulated arc therapy, and 6 to 8 mm for helical tomotherapy were added to the CTV to form the planning CTV. A dose of 50.4 Gy in 28 fractions was prescribed to the planning CTV, and a dose of 59 to 61 Gy was delivered to the PGTVnd with simultaneous integrated boost. For patients treated with pelvic RT, commonly used dose constraints of organs at risk for planning were as follows: spinal cord D0.1cc ≤ 45 Gy, bladder D50% ≤ 45 Gy, rectum D50% ≤ 45 Gy, and bowel D2cc ≤ 54 Gy. For patients treated with extended-field RT, additional constraints included kidney D30% ≤ 20 Gy and liver D30% ≤ 20 Gy. The constraints of bowel D50% were ≤ 20 Gy for patients receiving pelvic RT and ≤ 30 Gy for patients treated with extended-field RT.

For patients in both the pelvic RT and extended-field RT groups, intracavity brachytherapy was delivered with 192Ir, with 30 to 36 Gy in 5 to 7 fractions to point A.

The first-line regimen of CCRT was cisplatin (30–40 mg/m^2^ per week). Paclitaxel (60–80 mg/m^2^ per week) was administered for patients with renal failure.

### Follow-up and Evaluation of Toxicities

After treatment, follow-up examinations were performed every 3 months in the first 2 years, every 6 months during the third to fifth years, and once a year after 5 years. The routine follow-up examination included SCC antigen, gynecological examination, pelvic MRI, abdomen-, and chest-enhanced CT. The PET/CT and biopsy were performed for some patients with suspicious recurrence. Toxicities and complications were evaluated with Common Terminology Criteria for Adverse Events Version 3.0.

### Statistics

The end points of this study included OS, disease-free survival (DFS), distant failure, and para-aortic lymph node failure (PALNF). Lymph nodes proven by PET/CT or with a shorter diameter greater than 1 cm on CT images were considered as MLNs. Para-aortic lymph node failure was defined as MLNs in the area adjacent to the aorta and inferior vena cava, with an upper border of T12 and a lower border of the aortic bifurcation. Baseline demographic, clinical, and treatment characteristics of patients in the pelvic RT and extended-field RT groups were compared with χ^2^ test, continuity correction, and Fisher exact test before and after matching. Univariate and multivariate analyses were performed with the Cox regression model. Given the baseline characteristic differences between the 2 groups, we conducted propensity-score matching to identify the cohort of patients with similar baseline characteristics. Matching was carried out at a ratio of 1:1. The matching covariate included age, histology, FIGO stage, primary tumor size, pelvic MLNs, common iliac MLNs, bilateral pelvic MLNs, number of pelvic MLNs, and large pelvic MLNs (≥1.5 cm). Common iliac region was defined as the area adjacent to the common iliac vessels from the aortic bifurcation to the division of the common iliac artery to the external and internal iliac branches.^1^ Overall survival, DFS, distant failure, and PALNF rates were calculated with the Kaplan-Meier methods and compared between the pelvic RT and extended-field RT groups with the log-rank method before and after matching. Toxicities were compared between the 2 groups with χ^2^ test, continuity correction, and Fisher exact test, as appropriate. All statistical analyses were performed using SPSS (Version 22.0). A two-sided *P* value of less than 0.05 was considered statistically significant.

## RESULTS

A total of 833 stage IB–IVA patients with cervical cancer were treated with RT between January 2011 and December 2014. Fifty-five patients with para-aortic MLNs were excluded. At last, 778 patients met the inclusion criteria and were enrolled. Of them, 624 patients (80.2%) were treated with pelvic RT and 154 patients (19.8%) received extended-field RT. The baseline demographic, clinical, and treatment characteristics of patients in the pelvic RT and extended-field RT groups are shown in Table 1. Patients in the extended-field RT group had younger age (*P* = 0.002), more advanced stage (*P* < 0.001), and larger primary tumor size (*P* < 0.001), compared with patients in the pelvic RT group. More patients in the extended-field RT group had pelvic MLNs (*P* < 0.001). Patients in the extended-field RT group also experienced more extensive MLNs, including higher incidence of common iliac MLNs (*P* < 0.001) and ipsilateral/bilateral MLNs (*P* < 0.001), higher number of MLNs (*P* < 0.001), and larger pelvic MLNs (*P* < 0.001). In the pelvic RT and extended-field RT groups, PET/CT was conducted in 169 patients (27.1%) and 45 patients (29.2%, *P* = 0.595), respectively.

For the 154 patients treated with extended-field RT, the upper border of the extended field was at T12 in 53 patients, L1 in 56 patients, L2 in 40 patients, and L3 in 5 patients. For the 88 patients with pelvic MLNs and treated with extended-field RT, the median distance between the top the radiation field and the highest positive lymph node was 16.5 cm (range = 7–25.5 cm). For the 26 patients with common iliac lymph nodes, the median distance was 12 cm (range = 7–15.5 cm).

The median follow-up period for all cases was 37.5 months (range = 1.0–76.2 months). In the pelvic RT and extended-field RT groups, the median follow-up period was 38.0 months (range = 1.0–76.2 months) and 34.4 months (range = 2.2–72.4 months), respectively. During follow-up, 159 patients (20.4%) experienced treatment failure and 109 patients (14.0%) died. A total of 104 patients (13.4%) developed distant failure, with 81 patients (13.0%) in the pelvic RT group and 23 patients (14.9%) in the extended-field RT group. A total of 20 patients (2.6%) experienced PALNF, with 19 patients (3.0%) in the pelvic RT group and 1 patient (0.6%) in the extended-field RT group. The 3-year OS, DFS, local control, distant failure, and PALNF rates in the pelvic RT and extended-field RT groups were 88.5% and 79.6% (*P* = 0.003), 80.2% and 73.4% (*P* = 0.008), 91.1% and 83.5% (*P* = 0.005), 13.6% and 13.8% (*P* = 0.388), and 3.4% and 0.7% (*P* = 0.111), respectively.

In the 20 patients with PALNF, 9 received salvage irradiation. The median follow-up period (from the diagnosis of PALNF to death or lost to follow-up) was 9.5 months (range = 0.6–24.3 months). The median survival period was 10.3 months (95% confidence interval [CI] = 6.8–13.9 months). The 6-month and 1-year OS rates were 79.4% and 45.4%, respectively.

### Univariate and Multivariate Analysis

The results of univariate analysis are shown in the supplementary table, http://links.lww.com/IGC/A813. Patients treated with extended-field RT had worse OS (hazard ratio [HR] = 1.84, 95% CI = 1.22–2.79, *P* = 0.004) and DFS (HR = 1.59, 95% CI = 1.13–2.24, *P* = 0.009). Extended-field RT was not a significant factor of distant failure (HR = 1.23, 95% CI = 0.77–1.95, *P* = 0.389) and PALNF (HR = 0.22, 95% CI = 0.03–1.68, *P* = 0.145). Extended-field RT and other significant factors from the univariate analysis were analyzed by multivariate analysis. Considering it is clear that stage is associated with survival, we also included stage in the multivariate analysis, although it was not significant in predicting distant failure and PALNF in the univariate analysis. As shown in Table 2, extended-field RT was an independent prognostic factor of distant failure (HR = 0.49, 95% CI = 0.26–0.90, *P* = 0.023) and PALNF (HR = 0.012, 95% CI = 0.00–0.49, *P* = 0.019) in the multivariate analysis. However, it is not significant in predicting OS (HR = 0.84, 95% CI = 0.48–1.47, *P* = 0.546) and DFS (HR = 0.74, 95% CI = 0.47–1.16, *P* = 0.187).

### Propensity-Score Matching

With the use of propensity-score matching (1:1), 108 patients treated with extended-field RT were matched with 108 patients who underwent pelvic RT. As shown in Table 1, all baseline characteristics between the pelvic RT and extended-field RT groups were similar after matching. We also calculated the number of risk factors in the 2 groups after matching. Nine risk factors were included in the calculation, including the following: histology (non-SCC), FIGO stage (IIIB–IVA), primary tumor size (≥4 cm), pelvic MLNs, common iliac MLNs, bilateral pelvic MLNs, number of pelvic MLNs (≥3), large pelvic MLNs (≥1.5 cm), and age (≥65). In the extended-field RT and pelvic RT groups, the number of patients with 3 or more risk factors was 29 and 31 (*P* = 0.761), and the number of patients with 4 or more risk factors was 16 and 18 (*P* = 0.709), respectively. In the pelvic RT and extended-field RT group, PET/CT was conducted in 34 patients (31.5%) and 30 patients (27.8%, *P* = 0.551), respectively.

For the 216 patients, the median follow-up period after matching was 38.7 months (range = 2.2–76.2 months). Before and after matching, the events of death, treatment failure, distant failure, and PALNF in the extended-field RT group were 32 (20.8%) and 14 (13.0%, *P* = 0.102), 44 (28.6%) and 23 (21.3%, *P* = 0.184), 23 (14.9%) and 9 (8.3%, *P* = 0.108), and 1 (0.6%) and 0 (0, *P* = 1.000). After matching, the rates of all events decreased, although the decreases were not significant.

**FIGURE 1:**
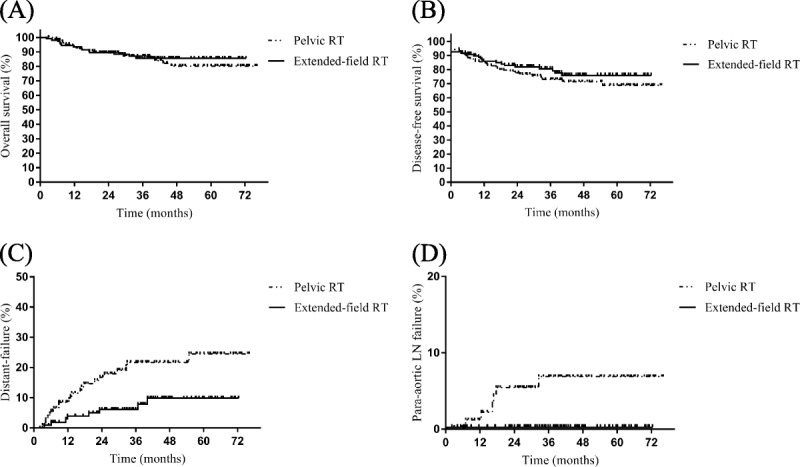
The overall survival (A), disease-free survival (B), distant failure (C), and para-aortic LN failure (D) of 212 patients with cervical cancer treated with pelvic RT and extended-field RT after propensity-score matching. LN indicates lymph node.

After matching, the 3-year OS, DFS, local control, distant failure, and PALNF rates in the pelvic RT and extended-field RT groups were 87.1% and 85.7% (*P* = 0.681, Fig. 1A), 71.0% and 80.6% (*P* = 0.199, Fig. 1B), 86.6% and 85.0% (*P* = 0.695), 21.7% and 7.0% (*P* = 0.016, Fig. 1C), and 6.6% and 0% (*P* = 0.014, Fig. 1D), respectively. The distant failure and PALNF rates in the extended-field RT group were significantly lower than those in the pelvic RT group. As shown in Figure 1B, the DFS rate in the extended-field RT group was higher than that of the pelvic RT group, although the difference was not significant.

**TABLE 1:**
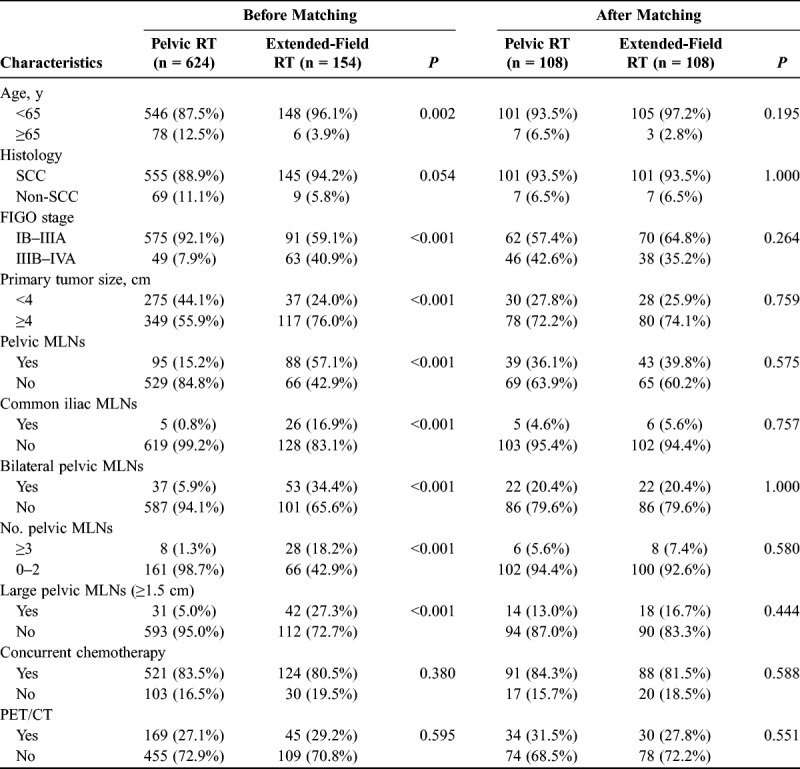
The Baseline Demographic, Clinical, and Treatment Characteristics of Patients in the Pelvic RT and Extended-Field RT Groups Before and After Propensity-Score Matching

**TABLE 2:**
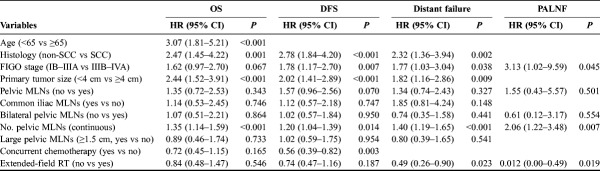
Results of Multivariate Analysis for Patients Treated With Pelvic RT and Extended-Field RT

### Toxicity

A total of 32 patients (4.1%) experienced grade 3 or greater chronic toxicities, including 22 patients (3.5%) in the pelvic RT group and 10 patients (6.5%) in the extended-field RT group (*P* = 0.097). As shown in Table 3, the incidence rates of all grade 3 or greater toxicities were not significantly different between the pelvic RT and extended-field RT groups. One patient with stage IIIB disease in the pelvic RT group died of intestinal obstruction. Another patient with stage IIB disease and pelvic MLNs developed acute renal failure after being administered 5.4 Gy in 3 fractions of extended-field RT and 1 cycle of cisplatin chemotherapy. The patient withdrew from treatment and died 1.5 months later.

**TABLE 3:**
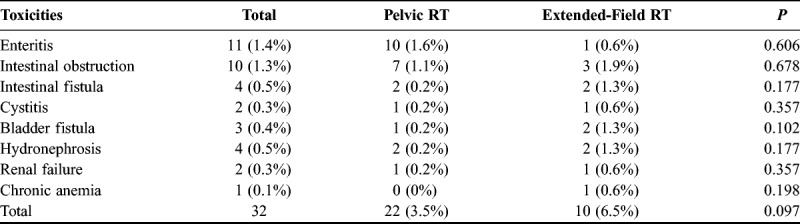
Chronic Toxicities of Patients Treated With Pelvic RT and Extended-Field RT

## DISCUSSION

The role of extended-field RT in patients with cervical cancer treated with CCRT has not been established at present. In a study from Saudi Arabia, 102 patients with FIGO stage IIB–IVA cervical cancer with negative para-aortic lymph nodes were randomized into pelvic RT-CCRT (50 patients) and extended-field RT-CCRT groups (52 patients). In the extended-field RT-CCRT group, a dose of 45 Gy was delivered to the para-aortic lymph node regions. Seventy-four patients were analyzed. Patients in the extended-field RT-CCRT group had improved para-aortic lymph node control, distant metastasis control, DFS, and OS.^11^ Lee et al^12^ reviewed 198 patients with locally advanced cervical cancer with pelvic MLNs and negative para-aortic lymph nodes. Of them, 80 patients underwent extended-field RT. Patients in the extended-field RT group had more advanced disease (including FIGO stage, common iliac MLNs, and number of pelvic MLNs). Compared with the pelvic RT group, the extended-field RT group exhibited better para-aortic lymph node recurrence-free survival, OS, cancer-specific survival, progression-free survival, and distant-free survival, especially for patients with common iliac MLNs and number of pelvic MLNs of 3 or greater.^12^ In some other studies, patients did not benefit from prophylactic extended-field RT. A study from Korea retrospectively analyzed 203 patients with locally advanced cervical cancer without para-aortic MLNs. Of them, 115 underwent pelvic RT and 88 received extended-field RT. No survival differences were observed between the 2 groups.^13^ In the study by Oh et al,^14^ 74 patients with cervical cancer were treated with pelvic RT and 52 patients underwent extended-field RT. The 10-year DFS, OS, and cumulative PALNF rates were not significantly different between the 2 groups.^14^ A study from Canada conducted multivariate analysis for 228 patients with cervical cancer and found that extended-field RT was not significantly associated with DFS, OS, and para-aortic relapse.^15^

In some of the retrospective studies described previously, the baseline characteristics of patients in the pelvic RT and extended-field RT groups were not balanced.^12^,^13^ The study from Korea suggested that extended-field RT did not have a significant effect. However, 67.0% of patients in the extended-field RT group had pelvic MLNs, compared with 29.6% in the pelvic RT group (*P* < 0.001). Patients in the extended-field RT group also experienced significantly more advanced stage and larger tumor size.^13^ With unbalanced baseline characteristics, the results had great bias. The baseline characteristics were also unbalanced between the 2 groups in our study. To reduce the bias, we used multivariate analysis and propensity-score matching. In multivariate analysis, extended-field RT was a significant factor of distant failure (HR = 0.49, 95% CI = 0.26–0.90, *P* = 0.023) and PALNF (HR = 0.012, 95% CI = 0.00–0.49, *P* = 0.019). After matching, the distant failure (*P* = 0.016) and PALNF (*P* = 0.014) rates for patients in the extended-field RT group were significantly lower than those in the pelvic RT group. These indicated the benefits of extended-field RT. It is regrettable that the improvement in distant failure and PALNF did not translate into an improvement in OS and DFS. As shown in Figure 1B, although it was not significant (*P* = 0.199), extended-field RT showed a trend in improving DFS. After 36 months, the OS curve of the extended-field RT group was also higher than that of the pelvic RT group (Fig. 1A). With a larger population and longer follow-up period, the differences may become significant.

The indication of prophylactic extended-field RT is also inconclusive. In previous studies, the inclusion criteria for extended-field RT included locally advanced cervical cancer,^11^,^13^ patients with pelvic MLNs,^12^,^14^,^16^ FIGO stage IB or IIA with primary tumor of 4 cm or greater, or FIGO stage IIB.^9^ As shown in Table 1, in our institute, patients treated with extended-field RT were more likely to have younger age, more advanced stage, larger primary tumor size, and more extensive pelvic MLNs. Various inclusion criteria may lead to different outcomes. This may be one of the reasons for the inconsistent results in previous studies.^11^–^15^ The aims of extended-field RT are to reduce distant failure and to improve DFS. Therefore, patients with a high risk of distant failure or treatment failure may benefit from extended-field RT. In recent years, there were several nomograms predicting treatment failure or survival in patients with cervical cancer treated with CCRT.^17^–^21^ In these studies, the risk factors of OS, DFS, and distant failure included adenocarcinoma,^17^,^19^,^21^ larger tumor size,^17^–^20^ parametrium involvement,^18^ advanced stage,^17^,^20^ MLNs,^17^,^20^,^21^ high SCC antigen level,^18^,^21^ etc. Nomograms based on these factors could accurately predict prognosis and treatment failure. In the future, if we could enroll patients based on these nomograms, we could accurately find patients who could benefit from extended-field RT, compared with single risk factors such as pelvic MLNs^12^,^14^,^16^ or large tumors.^9^

In most of the previous studies, the toxicities of extended-field RT were acceptable and not significantly higher than that of pelvic RT.^11^–^15^ In the present study, patients tolerated extended-field RT well. The incidence rate of grade 3 or greater chronic toxicities in the extended-field RT group was only 6.5%, and it was not significantly higher than that of patients in the pelvic RT group (3.5%, *P* = 0.097).

Neoadjuvant or adjuvant chemotherapies were other ways to improve the survival of patients with locally advanced cervical cancer besides prophylactic extended-field RT. Neoadjuvant chemotherapy has been evaluated in cervical cancer. It might reduce tumor volume and control micrometastatic disease. It was reported that neoadjuvant chemotherapy was associated with a high response rate in locally advanced cervical cancer.^22^,^23^ However, it is controversial whether neoadjuvant chemotherapy can improve the survival of patients with locally advanced cervical cancer compared with CCRT.^24^ An ongoing clinical trial, the INTERPLACE trial (NCT01566240), is a randomized phase III study evaluating neoadjuvant chemotherapy followed by CCRT for women with locally advanced cervical cancer compared with CCRT alone. In the present study, extended-field RT could only decrease distant failure. The OS rates were similar between the pelvic RT and extended-field RT groups. If the INTERPLACE trial finds that neoadjuvant chemotherapy could improve the survival of patients with cervical cancer, maybe we should reconsider the use of prophylactic extended-field RT.

As a retrospective study, there would have been inevitable biases in this study, although we have matched many factors. In our institute, most patients with a high risk of distant failure or treatment failure received extended-field RT, and we did not have enough high-risk patients for matching in the pelvic RT group. As a result, the proportion of high-risk patients was reduced after matching. For example, before matching, 26 patients (16.9%) in the extended-field RT group had common iliac MLNs. Only 5 patients (5.6%) with common iliac remained in the extended-field RT group after matching. Similarly, the proportion of patients with pelvic MLNs, bilateral pelvic MLNs, pelvic MLNs of 4 or greater, and large pelvic MLNs was also reduced after matching (Table 1). Treatment selection bias was another issue in this study. Even after propensity-score matching, patients in the extended-field RT group may have more advanced disease. However, even with more advanced disease, patients in the extended-field RT group showed lower distant failure and PALNF rates in the present study. Extended-field RT also showed a trend in improving DFS (Fig. 1B). If the baseline characteristics had been well balanced, the benefits of extended-field RT might be more obvious. In the present study, the numbers of events were comparatively small, especially for PALNF. With such a small number of events, the results for PALNF should be interpreted with caution. More randomized control trials are needed in the future to determine the efficacy of extended-field RT.

## CONCLUSIONS

This study indicated that patients with cervical cancer treated with definitive CCRT may benefit from prophylactic extended-field RT, because it was associated with reduced distant failure and PALNF, and showed a trend in improving DFS. The toxicities were similar between the 2 groups. More randomized control trials are needed in the future to determine the efficacy of extended-field RT.

## Supplementary Material

SUPPLEMENTARY MATERIAL
