# Supplementary material for: Prophylactic Extended-Field Irradiation for Patients With Cervical Cancer Treated With Concurrent Chemoradiotherapy: A Propensity-Score Matching Analysis
Source: Int J Gynecol Cancer. 2018 Aug 29;28(8):1584–91. doi: 10.1097/IGC.0000000000001344 (PMC6166702; doi:10.1097/IGC.0000000000001344)
Supplement: SUPPLEMENTARY MATERIAL [file igj-28-1584-s001.docx]

Supplementary table. Results of univariate analysis for patients treated with pelvic RT and extended-field RT

|  | OS | | DFS | | Distant failure | | PALNF | |
| --- | --- | --- | --- | --- | --- | --- | --- | --- |
| Variables | HR (95% CI) | p | HR (95% CI) | p | HR (95% CI) | p | HR (95% CI) | p |
| Age (<65 vs ≥65) | 2.11 (1.30-3.44) | 0.003 | 1.49 (0.96-2.29) | 0.073 | 1.38 (0.78-2.42) | 0.268 | 0.97 (0.23-4.20) | 0.972 |
| Histology (Non-SCC vs SCC) | 1.93 (1.15-3.25) | 0.013 | 2.15 (1.44-3.21) | <0.001 | 1.97 (1.17-3.31) | 0.011 | 1.10 (0.26-4.74) | 0.900 |
| FIGO stage (IB-IIIA vs IIIB-IVA) | 1.73 (1.08-2.76) | 0.022 | 1.76 (1.21-2.57) | 0.003 | 1.51 (0.92-2.48) | 0.107 | 1.61 (0.54-4.82) | 0.394 |
| Primary tumor size (<4 cm vs ≥4 cm) | 2.42 (1.55-3.78) | <0.001 | 2.21 (1.56-3.14) | <0.001 | 2.08 (1.34-3.22) | 0.001 | 2.13 (0.78-5.87) | 0.143 |
| Pelvic MLNs (Yes vs No) | 2.43 (1.65-3.57) | <0.001 | 2.20 (1.61-3.01) | <0.001 | 2.11 (1.42-3.15) | <0.001 | 2.88 (1.19-6.97) | 0.019 |
| Common iliac MLNs (Yes vs No) | 3.57 (1.96-6.51) | <0.001 | 2.71 (1.57-4.69) | <0.001 | 3.53 (1.89-6.60) | <0.001 | 3.04 (0.70-13.11) | 0.136 |
| Bilateral pelvic MLNs (Yes vs No) | 2.91 (1.88-4.50) | <0.001 | 2.47 (1.71-3.57) | <0.001 | 2.23 (1.38-3.61) | 0.001 | 2.85 (1.03-7.84) | 0.043 |
| Number of pelvic MLNs (Continuous) | 1.40 (1.29-1.53) | <0.001 | 1.30 (1.20-1.40) | <0.001 | 1.35 (1.23-1.47) | <0.001 | 1.36 (1.12-1.64) | 0.002 |
| Large pelvic MLNs (≥ 1.5 cm, Yes vs No) | 2.43 (1.50-3.95) | <0.001 | 2.09 (1.38-3.15) | <0.001 | 1.91 (1.12-3.26) | 0.017 | 1.83 (0.54-6.23) | 0.337 |
| Concurrent chemotherapy (Yes vs No) | 0.62 (0.40-0.95) | 0.029 | 0.67 (0.46-0.96) | 0.028 | 0.70 (0.44-1.11) | 0.126 | 1.10 (0.32-3.76) | 0.876 |
| Extended-field RT (Yes vs No) | 1.84 (1.22-2.79) | 0.004 | 1.59 (1.13-2.24) | 0.009 | 1.23 (0.77-1.95) | 0.389 | 0.22 (0.03-1.68) | 0.145 |

Abbreviation: CI = confidence interval; DFS = disease-free survival; FIGO = International Federation of Gynecology and Obstetrics; HR = hazard ratio; MLNs = metastatic lymph nodes; OS = overall survival; PALNF = para-aortic lymph node failure; RT = radiation therapy; SCC = squamous cell carcinoma
